# The Effect of Freeze-Thaw Cycles on Gene Expression Levels in Lymphoblastoid Cell Lines

**DOI:** 10.1371/journal.pone.0107166

**Published:** 2014-09-05

**Authors:** Minal Çalışkan, Jonathan K. Pritchard, Carole Ober, Yoav Gilad

**Affiliations:** 1 Department of Human Genetics, The University of Chicago, Chicago, Illinois, United States of America; 2 Departments of Genetics and Biology and Howard Hughes Medical Institute, Stanford University, Stanford, California, United States of America; The George Washington University, United States of America

## Abstract

Epstein-Barr virus (EBV) transformed lymphoblastoid cell lines (LCLs) are a widely used renewable resource for functional genomic studies in humans. The ability to accumulate multidimensional data pertaining to the same individual cell lines, from complete genomic sequences to detailed gene regulatory profiles, further enhances the utility of LCLs as a model system. However, the extent to which LCLs are a faithful model system is relatively unknown. We have previously shown that gene expression profiles of newly established LCLs maintain a strong individual component. Here, we extend our study to investigate the effect of freeze-thaw cycles on gene expression patterns in mature LCLs, especially in the context of inter-individual variation in gene expression. We report a profound difference in the gene expression profiles of newly established and mature LCLs. Once newly established LCLs undergo a freeze-thaw cycle, the individual specific gene expression signatures become much less pronounced as the gene expression levels in LCLs from different individuals converge to a more uniform profile, which reflects a mature transformed B cell phenotype. We found that previously identified eQTLs are enriched among the relatively few genes whose regulations in mature LCLs maintain marked individual signatures. We thus conclude that while insight drawn from gene regulatory studies in mature LCLs may generally not be affected by the artificial nature of the LCL model system, many aspects of primary B cell biology cannot be observed and studied in mature LCL cultures.

## Introduction

The use of lymphoblastoid cell lines (LCLs) in human genetics studies has been controversial for some time. On the one hand, LCLs are a renewable resource that provides investigators with the nearly unique opportunity to perform in-depth studies of molecular and complex phenotypes using the same collection of samples [1]–[4]. On the other hand, the transformation that immortalizes the LCLs, through the infection of primary B cells with EBV, is known to result in certain artifacts [2], [5]. The question, therefore, is to what extent LCLs faithfully represent the biological process of primary tissues, specifically of their progenitors, primary B cells.

Thousands of published studies have used LCLs to address a wide range of questions in human biology, including studies of gene regulatory mechanisms [3], [4], [6]–[14], drug toxicity [15], [16], response to stress [17], [18], and case control studies of human diseases [19], [20]. Yet, perhaps surprisingly, the literature specifically addressing the extent to which LCLs are a faithful model system is relatively scarce. For example, LCLs have been extensively used to study the genetic and mechanistic basis for variation in gene regulatory phenotypes [3], [4], [6]–[14]. We have gained much insight from regulatory studies in LCLs and replication across studies has been satisfactory, including replication of results based on data from LCLs in studies that used primary tissues [3], [4], [6]–[14]. Indeed, there is little concern that the large body of work on gene regulation using LCLs as a model system has resulted in erroneous observations or insights. Yet, it is also the case that we still do not fully understand to what extent gene regulatory programs in LCLs truly reflects those of primary tissues.

In a previous study [21], we directly addressed the effect of EBV transformation on gene expression profiles in newly immortalized LCLs. To do so, we isolated primary B cells from six individuals (three males and three females) and established six LCL cultures from each individual by performing independent replicates of EBV transfections. We then collected gene expression data from the primary B cells as well as from all of the cultured LCLs. We found that the expression levels of thousands of genes were significantly altered following EBV transformation. Yet, the genome-wide gene expression profiles of the six independently immortalized LCLs from each individual were more correlated to each other than to gene expression profiles of LCLs across individuals. In other words, in the previous study [21], we have demonstrated that gene expression levels in newly established LCLs maintain a strong individual component. Based on these observations we argued that newly established LCLs are a useful system for studies of the genetic basis for variation in gene expression levels. Yet, we also noted that, almost without exception, gene regulatory studies in LCLs are not using newly transformed cultures but rather they use LCLs that experienced quite a few freeze-thaw cycles, namely more mature LCLs.

Since the publication of our study on the effects of EBV transformation on gene expression levels, three years ago, we have continued to culture the LCLs that were established for that study. We carried out six freeze-thaw cycles and collected gene expression data throughout this time course. The data we collected allowed us to address additional questions regarding the usefulness of mature LCL cultures for studies of individual variation in gene expression.

## Materials and Methods

### Ethics statement

Unpurified buffy coats were purchased from Research Blood Components (http://www.researchbloodcomponents.com/index.html) between October 2009 and January 2010. Research Blood Components obtained an IRB approved consent form from each donor giving permission to collect their blood and use or sell it at Research Blood Components's discretion, for research purposes.

### Sample collection

We obtained unpurified buffy coats from six unrelated healthy individuals of Caucasian ethnicity (age range: 20–45). We isolated CD20+B cells and established six independent cultures of LCLs between October 2009 and January 2010, as previously described [21]. From each of these samples, we previously extracted RNA and DNA, obtained genome wide gene expression data using Illumina HumanHT-12 v3 Expression BeadChip arrays and quantified relative EBV and mtDNA copy numbers [21]. We refer to data from these experiments as ‘cycle 0’ to acknowledge the fact that the LCLs from this cycle were newly established and therefore not frozen/thawed.

Between February 2011 and October 2012, we thawed each of these LCL cultures every 3 months and cultured them until obtaining ∼10 million cells (February 2011 = cycle 1, June 2011 = cycle 2, October 2011 = cycle 3, February 2012 = cycle 4, June 2012 = cycle 5, and October 2012 = cycle 6). Due to contamination, we lost two LCL cultures from individual three at cycle 1, one LCL culture from individual four at cycle 3, and one LCL culture from individual five at cycle 4.

### RNA extraction and genome wide gene expression profiling

We extracted RNA from each of the LCL cultures at every other freeze-thaw cycle (cycle 2, cycle 4, cycle 6) using QIAGEN RNeasy Plus Mini Kit. We measured the concentration and the quality of RNA using Agilent 2100 Bioanalyzer and obtained genome wide gene expression data using Illumina HumanHT-12 v4 Expression BeadChip arrays. The University of Chicago Functional Genomics Core performed the cDNA synthesis, labeling, and hybridization of RNA to the microarrays. At each hybridization batch we included a subset of RNA samples that were also hybridized in a previous batch. This allowed us to effectively consider the batch effect on gene expression profiles. Our final dataset included genome wide gene expression data from 187 samples collected at 4 different time points (GEO accession number: GSE58942).

We used the Bioconductor software package lumi for low-level gene expression data analysis. Among the 48,804 probes that were targeted by Illumina HumanHT-12 v3 Expression BeadChip arrays and 47,231 probes that were targeted by Illumina HumanHT-12 v4 BeadChip arrays, 24,314 overlapping probes passed our probe quality control check (probes that mapped to unique Ensembl gene IDs, probes that did not contain any HapMap SNPs with minor allele frequency >0.01 in the CEU population, probes that targeted autosomal chromosomes). We performed all subsequent analyses using data from 12,972 (of the 24,314) probes that were detected as expressed (probes with a detection *P* value <0.05 in at least 25% of the 187 samples). We log_2_-transformed and rank-invariant normalized the probe intensity data, and then used the median probe intensity level per gene as the estimated gene expression level of 10,313 Ensembl genes. We performed principal components analysis (PCA) using pair-wise sample covariance matrix.

### DNA extraction and quantification of EBV and mtDNA copy number

We used QIAamp DNA Blood Mini Kit to extract DNA at every other freeze-thaw cycle (cycle 2, cycle 4, cycle 6). We quantified DNA concentration using Nanodrop ND-100 Spectrophotometer. To be able to perform inter plate normalization across all qPCR plates, we re-quantified the relative EBV and mtDNA copy numbers of the cycle 0 LCLs. We determined the relative EBV and mtDNA copy numbers of the LCLs using the 2^(-Delta Delta Ct)^ method [22], as described [21]. Briefly, for each LCL sample we used Applied Biosystems TaqMan pre-designed assays to amplify 87 bp fragment of the host nuclear-*RPPH1* gene (*RNase P* assay), 72 bp fragment of the EBV-*IR1* gene, and 151 bp fragment of the host mt-*CYB* gene. *RNase P* assay was used to serve as an internal reference gene to normalize the qPCRs for the amount of nuclear DNA added. A calibrator LCL DNA was included in each qPCR plate for inter-plate normalization.

### Calculation of correlation between gene expression levels and EBV copy numbers

For each gene in our data, we calculated the absolute value of Spearman rank correlation coefficient between gene expression levels and EBV copy numbers within each freeze-thaw cycle. In order to assess the significance, we shuffled EBV copy numbers (3 times) and repeated our calculation. We estimated the FDR by comparing the observed distribution of the Spearman rank correlation coefficients to the null distribution; we calculated the Spearman rank correlation coefficient i corresponding to FDR 1% threshold as Probability (|Spearman rank correlation coefficient permuted| <i)/Probability (|Spearman rank correlation coefficient observed| <i)  = 0.01. Among each of the three comparisons, we selected the most conservative FDR 1% threshold to assess significance.

### Calculation of pair-wise sample correlations and coefficient of variation of gene expression

Within each freeze-thaw cycle, we calculated within-individual pair-wise Pearson correlation coefficients of gene expression profiles. Similarly, within each freeze-thaw cycle we randomly picked one LCL culture of each individual and calculated between-individual pair-wise Pearson correlation coefficients of the gene expression profiles.

To estimate gene expression variation, we calculated the between-individual coefficient of variation (CV) of gene expression for each gene in our data. This analysis was performed using one randomly picked LCL of each individual within each freeze-thaw cycle.

### KEGG pathway enrichment analyses

The web-based DAVID functional annotation bioinformatics database [23] was used to test for enrichment of Kyoto Encyclopedia of Genes Genomes (KEGG) pathways among each gene set of interest. 10,313 genes that were detected as expressed in our data were used as the background gene set in all enrichment analyses.

## Results

To evaluate the effects of freeze-thaw cycles on gene expression levels in LCLs, we collected whole genome gene expression data (using microarrays; see Methods) from primary CD20+B cells, from their corresponding newly established LCLs (cycle 0), and from the same LCLs after two (cycle 2), four (cycle 4), and six (cycle 6) cycles of freezing and thawing. We refer to the cultures that experienced at least one freeze-thaw cycle as ‘mature LCLs’. A schematic of the study design and the microarray hybridization strategy is shown in Figure S1 in Appendix S1. The details regarding the cell culture time course are available in Table S1 in Appendix S1. Following quality control analysis (see Methods) we obtained estimates of gene expression levels for 10,313 genes across CD20+B cells and their corresponding LCLs throughout all cycles.

**Figure 1:**
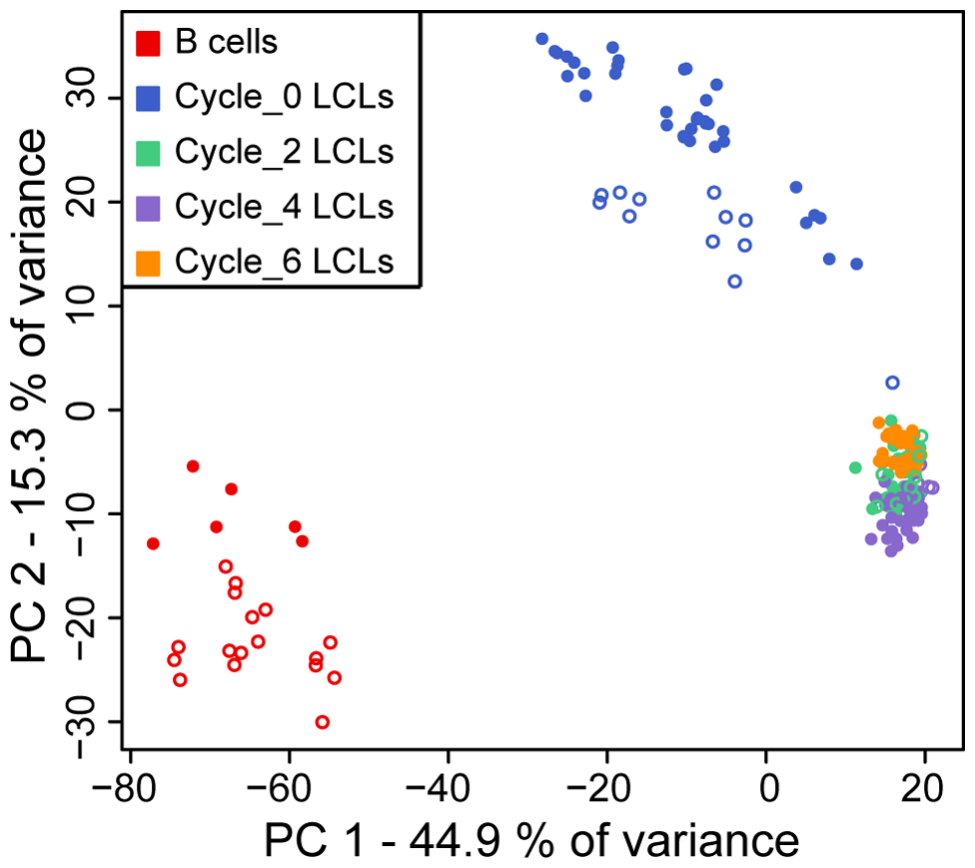
PCA of gene expression data. PCA was performed using pair-wise sample covariance matrix of 187 samples and applied to the genome-wide gene expression data (10,313 genes detected as expressed). Filled circles correspond to original hybridization and empty circles correspond to technical hybridization replicates (samples that were hybridized in more than one array batch).

As a first step, we performed PCA to identify the major sources of variation in the gene expression data (Figure 1). This analysis confirmed our previous observations of a substantial difference in overall gene expression profiles between primary B cells and newly established LCLs (Figure S2 in Appendix S1; T test on the pair wise Euclidean distance within and between classes of cells; *P*<2.2×10^−16^). It also indicated that there is a marked difference in gene expression profiles between newly established LCLs on the one hand, and LCLs that experienced one or more cycles of freezing and thawing on the other hand (Figures 1 and S2 in Appendix S1). In what follows, we refer to this property of the data as the effect of freeze-thaw cycles on gene expression in LCLs, though it is important to note that our study design does not allow us to distinguish between the specific effects related to freezing and thawing the LCLs and those of continued passaging of the same cell culture.

### The freeze-thaw effect on EBV and mtDNA copy numbers

**Figure 2:**
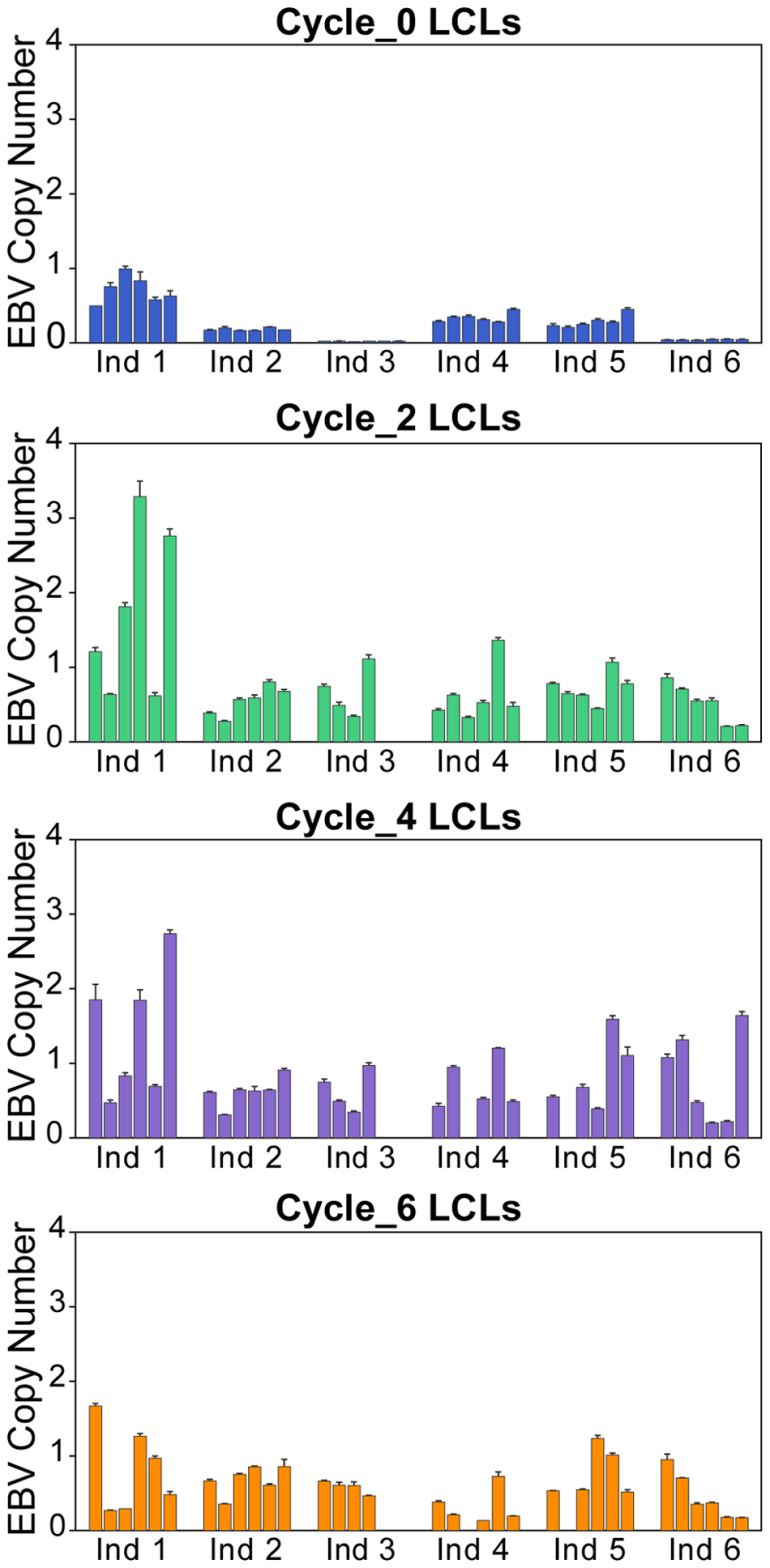
Relative EBV copy numbers in LCLs from six individuals. Error bars indicate the standard error of the mean.

Infection of quiescent (G_0_) B cells with Epstein Barr Virus (EBV) leads to establishment of LCLs *in vitro*. Previous studies [21], [24] showed that EBV viral load in LCLs is a variable trait that contributes to the variation in gene expression levels. We therefore first considered a difference in EBV copy numbers as a driver of the overall difference in gene expression profiles as the LCLs were cultured through freeze-thaw cycles. To do so, we used qPCR to estimate relative EBV copy numbers in all LCL cultures in our study (see Methods). We found a clear difference in EBV copy numbers between newly established LCLs (cycle 0) and LCLs that were frozen and thawed at least twice, but relatively little difference in EBV copy numbers between mature LCLs that were frozen and thawed either 2, 4, or 6 times (Figure 2 and Table S2 in Appendix S1). We next aimed to identify genes whose expression was associated with variation in EBV viral load. Because EBV copy number was essentially confounded with the freeze-thaw cycle, we tested correlations between EBV copy number and gene expression levels within each freeze-thaw cycle separately. In newly established LCLs, the expression level of 16.7% of genes (1,719 out of 10,313) showed significant correlation with EBV copy number (Figure S3 in Appendix S1, Appendix S2; see Methods), and these were enriched for genes involved in DNA replication pathways, cell cycle, and DNA repair (Table S3 in Appendix S1), as expected [25]. In the following freeze-thaw cycles, the correlations between EBV copy number and gene expression levels were no longer significantly different than what is expected by chance alone (Figure S3 in Appendix S1). We performed a similar analysis with respect to the copy number of mitochondrial DNA (mtDNA), which is often taken as an indication of the progression of EBV-mediated B-cell transformation [26]. We again observed a significant increase in mtDNA copy number following the first freeze-thaw cycle, but little variation in subsequent cycles (Figure S4 and Table S4 in Appendix S1).

### Effect of freeze-thaw cycles on individual signatures of gene expression profiles

**Figure 3:**
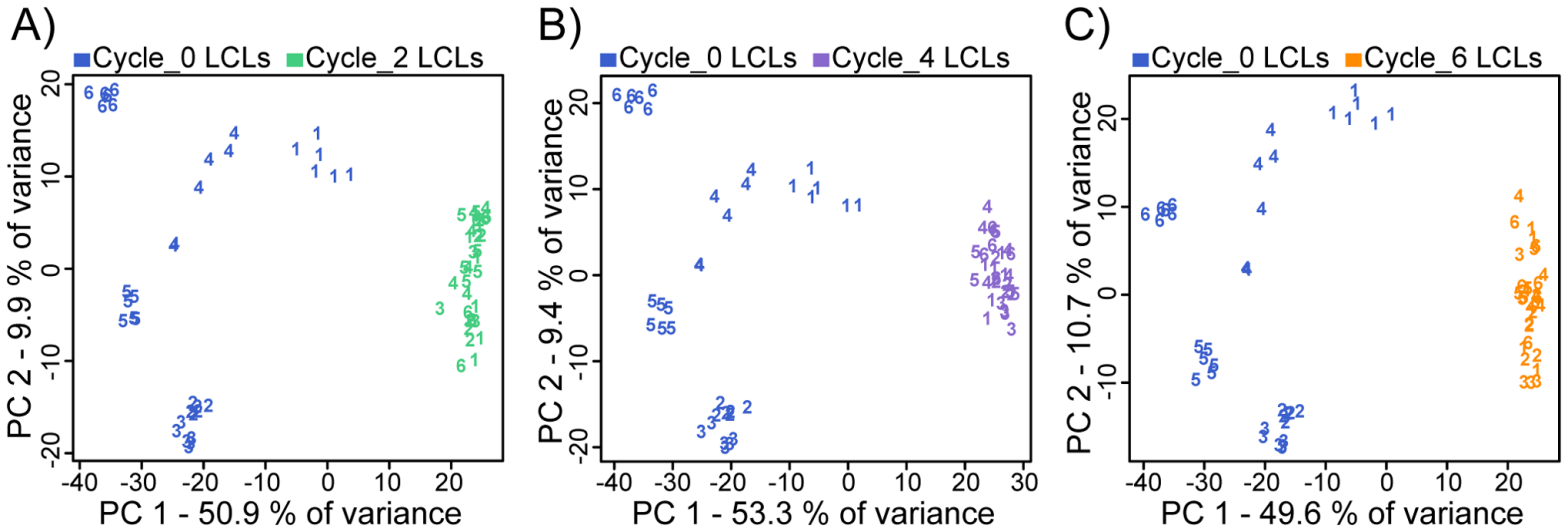
Individual effect on gene expression patterns. PCA was applied to the genome-wide expression data of **A**) cycle 0 and cycle 2 **B**) cycle 0 and cycle 4 **C**) cycle 0 and cycle 6 LCL cultures. Numbers correspond to the individual origin of the culture (of the six individuals).

As we mentioned in the introduction, in our previous study [21] we found that gene expression profiles of newly established LCLs retain a strong individual signature. Once the LCLs have experienced one or more freeze-thaw cycles, however, we found that gene expression profiles from independently established mature LCLs from the same individual no longer exhibit distinctively high correlations (Figure 3 and Figure S5 in Appendix S1). Variation in EBV and mtDNA copy numbers across LCLs do not seem to explain the drastic decrease in individual signatures of gene expression profiles of mature LCLs (Figure S6 in Appendix S1). Instead, the reason for this seems to be a significant increase in overall correlation in gene expression levels between LCLs, regardless of the individual origin of the culture (Figure S7 and Table S5 in Appendix S1). In other words, once newly established LCLs undergo freeze-thaw cycles, the individual specific gene expression signatures become much less pronounced as the gene expression levels in LCLs from different individuals converge to a more uniform profile, which reflects a mature transformed B cell phenotype. Mostly, these nearly uniform LCL gene expression profiles are achieved already after the second freeze-thaw cycle, with little additional change in subsequent cycles.

**Figure 4:**
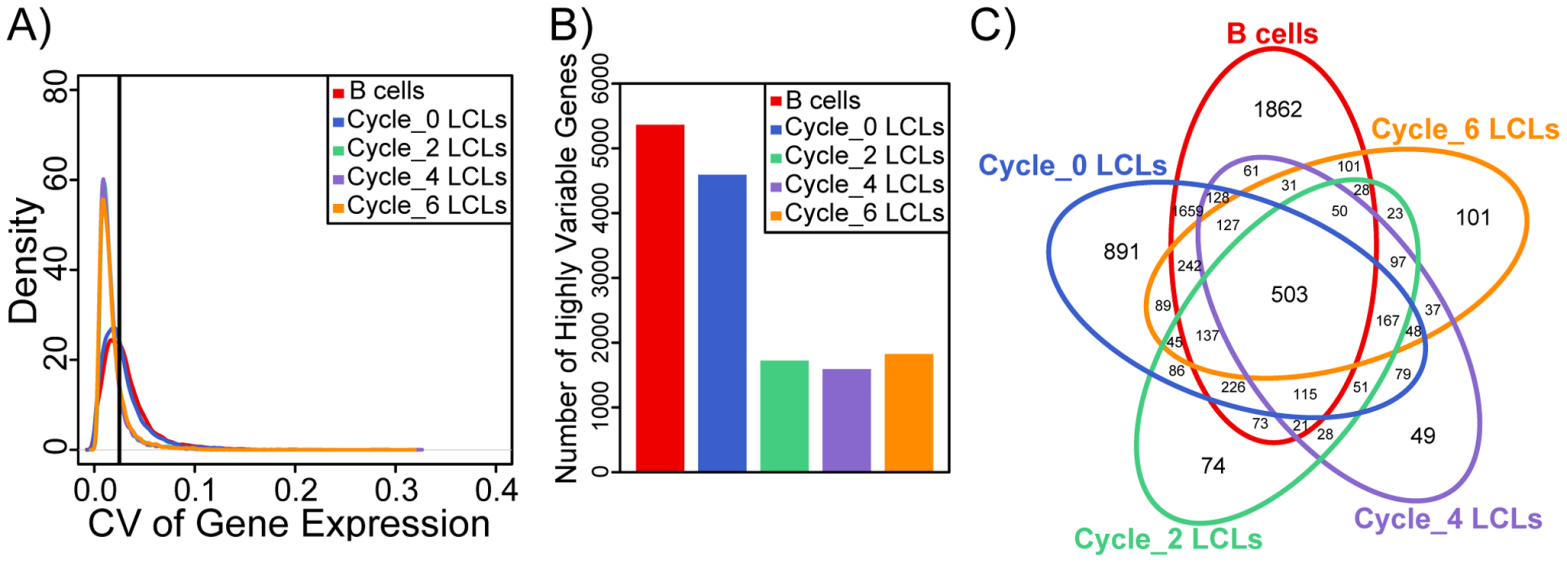
Gene expression variation in mature LCLs can observe only a fraction of the inter-individual variation in gene expression levels that exists in the primary B cells. **A**) Density distributions of the coefficient of variation (CV) of gene expression between-individuals within primary B cells and within LCLs of each freeze-thaw cycle. Black vertical line designates the arbitrarily chosen threshold of CV of 0.025. **B**) Bar plots showing the numbers of genes classified as having ‘highly variable’ expression patterns in the primary B cells, cycle 0, cycle 2, cycle 4, and cycle 6 LCLs. **C**) Venn diagram of the overlaps in genes with ‘highly variable’ expression patterns across primary B cells, cycle 0, cycle 2, cycle 4, and cycle 6 LCLs. We also plotted an equivalent figure based on residual data after regressing out mtDNA and EBV copy numbers; see Figure S10 in in Appendix S1.

The severe reduction in individual signatures of gene expression in mature LCLs seem to be counter-intuitive given numerous reports (including from our own group) of successful eQTL mapping efforts in mature (sometimes decades old) LCLs across individuals. We reasoned that for our observations to be consistent with earlier studies, previously identified eQTLs must be over-represented among the relatively few genes that maintain high level of inter-individual variation in gene expression levels in mature LCLs. To examine this, we first calculated the coefficient of variation (CV) of gene expression between-individuals within each cell type/freeze-thaw cycle (Figure 4A, Appendix S2). Based on the entire distributions of CV (Figure 4A), we arbitrarily designated genes with CV of more than 0.025 as those that maintained individual signature of gene expression in the mature LCLs. We refer to the expression patterns of these genes as ‘highly variable’ among individuals. As expected, we found a large number of ‘highly variable’ genes in the primary B cells (5,364 genes) and in newly established LCLs (4,593 genes), and many fewer such genes in LCLs that have experienced two or more freeze-thaw cycles (1,724, 1,592, and 1,826, and LCLs from cycles 2, 4, and 6, respectively; Figure 4B and Figure 4C). The results of KEGG pathway enrichment analyses considering the ‘highly variable’ genes across all cell types/freeze-thaw cycles or only the ‘highly variable’ genes in a specific cell type or freeze-thaw cycle are included in Tables S6 and S7 in Appendix S1.

**Figure 5:**
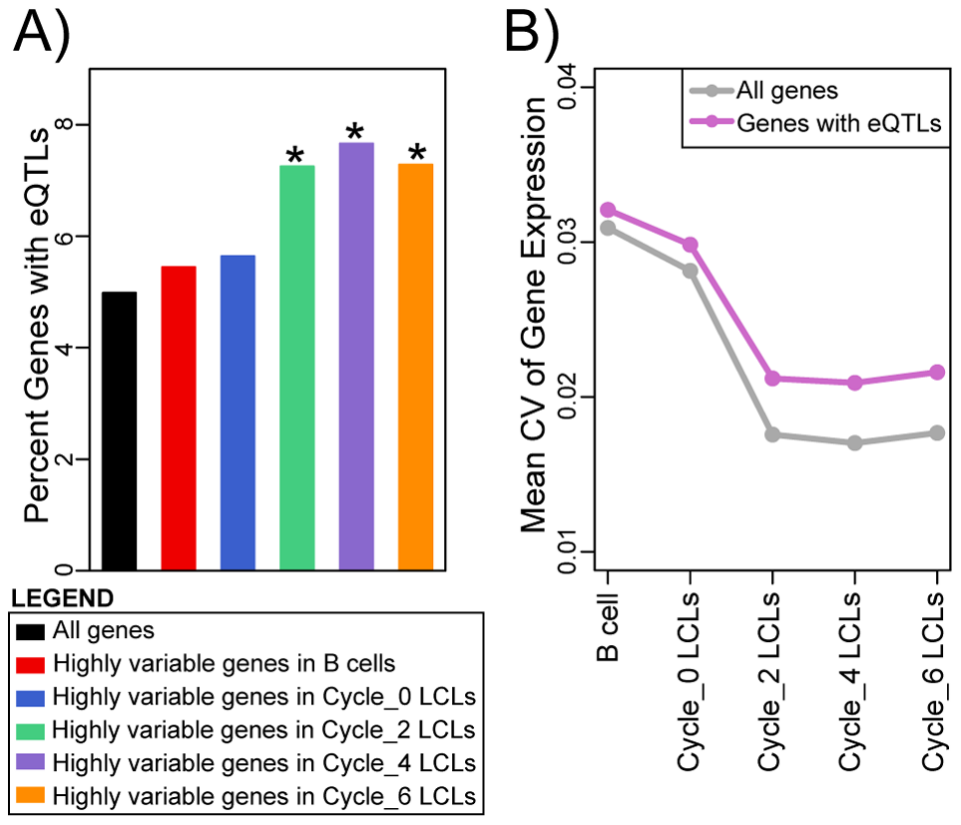
Previously identified eQTLs are enriched among the genes whose regulations in mature LCLs maintain marked inter-individual variation. **A**) Bar plots showing the percent of genes with eQTLs (as identified in LCL-Study 5) among all genes detected as expressed and among genes with ‘highly variable’ expression patterns in B cells, cycle 0, cycle 2, cycle 4, and cycle 6 LCLs. Asterisks indicate that the null hypothesis of no difference in proportion of genes with eQTLs relative to that in all expressed genes was rejected at *P* values <8.3×10^−4^ (Bonferroni corrected significance threshold). Results pertaining to all twelve eQTL studies are included in Figure S8 and Table S8 in Appendix S1. **B**) Mean coefficient of variation (CV) of gene expression within each cell type/freeze-thaw cycle for all the genes detected as expressed and for genes with eQTLs (as identified in LCL Study-5). Results for all twelve eQTL studies are included in Figure S9 and Table S9 in Appendix S1.

We next integrated our results with those of seven previously published eQTL studies in LCLs [3], [6]–[11], one study in cortex [27], one in liver [28], one in fibroblasts [7], one in T cells [7], and one in monocytes [29] (Table 1). Generally speaking, we found only weak evidence for enrichment of genes with eQTLs among genes with ‘highly variable’ expression patterns in the primary B cells or in the newly established LCLs (Figure 5A, Figure S8 and Table S8 in Appendix S1). This observation is expected given that in all of the studies we considered, the number of detected eQTLs was low compared to the number of expressed genes. In contrast, we found moderate to strong evidence (depending on the eQTL study considered; Figure 5A, Figure S8 and Table S8 in Appendix S1) for enrichment of eQTLs among genes with ‘highly variable’ expression patterns in the mature LCLs, throughout the timecourse (*P*<8.3×10^−4^). The enrichment was stronger for eQTLs that were originally found in LCLs, other blood cells types, or fibroblasts, and inconclusive when we considered eQTLs that were identified using gene expression data from liver or brain (Figure 5A, Figure S8 and Table S8 in Appendix S1). Considered from a different perspective, we observed that the average CV of genes with *cis*-eQTLs is in general higher than that of all the genes detected as expressed in our study (Figure 5B, Figure S9 and Table S9 in Appendix S1). Moreover, the difference in CV between genes with eQTLs and that of all the expressed genes is more pronounced in mature LCLs than in the B cells or newly established LCLs (Figure 5B, Figure S9 and Table S9 in Appendix S1).

**Table 1:** The list of eQTL studies whose results were downloaded from the Pritchard Lab eQTL browser (eqtl.uchicago.edu).

eQTL study name	Number of Ensembl genes with cis eQTLs	Number of Ensembl genes with cis eQTLs that were detected as expressed in our study	Reference
LCL-Study 1	438	325	[3]
LCL-Study 2	794	561	[6]
LCL-Study 3	442	318	[7]
LCL-Study 4	1084	790	[8]
LCL-Study 5	830	514	[9]
LCL-Study 6	1894	1564	[10]
LCL-Study 7	778	614	[11]
Cortex-Study	68	38	[27]
Liver-Study	2057	1155	[28]
Fibroblast-Study	427	286	[7]
T cell-Study	430	273	[7]
Monocyte-Study	2510	1714	[29]

### Recapitulating biologically meaningful gene expression patterns

Finally, we examined whether we could recapitulate an individual signature of gene expression patterns in mature LCLs by focusing exclusively on the genes with ‘highly variable’ expression patterns, for which eQTLs were previously identified by studies shown in Table 1. Across cell types and the entire timecourse experiment, we classified 503 genes as ‘highly variable’ (Figure 4C). Of these, 263 were associated with an eQTL in at least one study (see Table S10 in Appendix S1 for the number of ‘highly variable’ genes with eQTLs in each study).

Focusing on a reduced data set of one primary B cell and one randomly chosen LCL from each individual in each freeze-thaw cycle, we performed PCA on either all expressed genes, or by considering the expression data from only the ‘highly variable’ genes that have eQTLs (Table S10 in Appendix S1). We used linear regression to test for an association between three variables (cell type, freeze-thaw cycle, individual) and the axis-scores of the first five principal components. Using this approach, we found that the major sources of variation in the genome-wide data are ‘cell type’ and ‘freeze-thaw cycle’ as we mentioned earlier (Table S11A in Appendix S1). In the genome-wide data, individual effect was not associated with any of the first five principal components. In contrast, when we focused on the ‘highly variable’ genes with eQTLs, we found that individual effect is one of the major sources of variation in the data (i.e., individual effect is significantly correlated with either the first or the second principal components; Table S11B and Table S11C in Appendix S1).

## Discussion

We found that gene expression profiles in newly established LCLs are quite different than that of mature LCLs. Newly established LCLs still reflect a large component of the individual-specific variation in gene expression seen in primary B cells. In mature LCLs, however, much of this variation in gene expression is absent. In general, we found much less variation in gene expression levels among mature LCLs than among newly established LCLs, regardless of the individual origin of the culture. It would seem that once the LCL culture experiences freeze-thaw cycles, an acute selection process occurs, and the culture converges to a much more homogeneous gene expression profile (compared with the regulatory phenotypes of newly established LCLs), that of a mature self renewing LCL culture.

Our observations suggest that studies of gene expression variation in mature LCLs can observe only a fraction of the inter-individual variation in gene expression levels that exist in the primary tissue. Much of the naturally occurring gene expression variation, which could be observed in primary B cells and even – based on our findings – in newly established LCLs, is absent in mature LCL cultures. Yet, our results also suggest that the associations between genotype and gene expression levels that are found in mature LCLs likely truly reflect those that exist in primary cell types (i.e., genes that have eQTLs in T cells, monocytes, and fibroblasts are enriched among ‘highly variable’ genes in mature LCLs). Thus, we conclude that on the one hand, findings and insight drawn from gene regulatory studies in mature LCLs are unlikely to be affected by the artificial nature of the LCL model system and reflect a residual of the biology of primary B cells. On the other hand, significant reduction in between-individual gene expression variation suggests that many aspects of primary B cell biology cannot be observed and studied in mature LCL cultures. If we borrow the terminology of type I and type II error to express our conclusions, we argue that the false positive rate for findings in LCLs is expected to be low (we predict that most significant observations could be replicated in primary tissues), but the false negative rate is expected to be high (much of the variation that exist in primary tissues cannot be observed in mature LCLs).

An alternative explanation for our observations is that much of the individual variation in gene expression found in primary B cells is the result of environmental effects (or gene by environment interactions), as suggested previously [30]. In mature LCLs, the absence of individual-specific environmental effects results in a much more uniform gene expression profile. If this explanation is correct, the power to detect eQTLs in mature LCLs may actually be higher than in primary B cells, where environmental effects may mask much of the genetic effects on gene regulation. In other words, both the false positive and false negative rates associated with identifying eQTLs in mature LCLs may be low. Indirect support for this alternative explanation, and its implications, can be found by considering the few published eQTL studies in primary tissues [31]–[33]. It does not seem that many more eQTLs were identified in such studies than in studies using mature LCLs.

However, it should be noted that most eQTL studies in primary tissues used complex samples rather than samples consisting of a single pure cell type. It is therefore possible that in pure cultures of primary cells one could identify many more eQTLs than in either LCLs or complex tissues. In addition, our observation that inter-individual variation in gene expression profiles of newly established LCLs recapitulates much of the individual variation observed in primary B cells, argues against the alternative explanation based on environmental effects. The newly established LCLs were cultured for a minimum of three weeks (most lines were cultured for more than a month) prior to RNA extraction. It therefore seems unlikely that much of the environmental effects on gene expression in the primary B cells would persist in newly established LCLs.

Our current understanding of the genetic and mechanistic basis for gene regulatory variation in humans relies heavily on studies in mature LCLs. Our observations suggest that much of the insight we have should indeed reflect the biology in primary tissues and that as such, LCLs have been a useful model system. Early studies using LCLs focused on moderate sample sizes (60–90 individuals) and typically identified proximal (putatively *cis*) eQTLs for roughly 10% of genes. More recent studies using LCLs include much larger sample size (several hundreds to several thousands). The larger sample size allows for the identification of many more associations of genotype and gene expression levels; these studies practically identify a *cis* eQTL for every expressed gene. The effect sizes of many of the eQTLs identified with these larger sample sizes is rather small. In our study, with LCLs from only 6 individuals, we are not able to observe such individual differences in gene expression levels, even in the primary B cells. Nevertheless, a generalization of our observations suggests that the eQTLs identified in the large studies in mature LCLs should reflect true individual regulatory variation. Yet, our observations also suggest that with a different system, one in which much of the gene expression variation is not so drastically reduced, a similar number of eQTLs may be identified with much smaller sample sizes.

## Supporting Information

Appendix S1 **Supplementary appendix including Figures S1 to S10 and Tables S1 to S11.**
(PDF)

Appendix S2 **Table showing EBV-gene expression correlation coefficients within each freeze-thaw cycle and the coefficient of variation of gene expression within B cells and within each freeze-thaw cycle.**
(XLSX)
