# Supplementary material for: The Effect of Freeze-Thaw Cycles on Gene Expression Levels in Lymphoblastoid Cell Lines
Source: PLoS One. 2014 Sep 5;9(9):e107166. doi: 10.1371/journal.pone.0107166 (PMC4156430; doi:10.1371/journal.pone.0107166)
Supplement: Appendix S1 — Supplementary appendix including Figures S1 to S10 and Tables S1 to S11. (PDF) [file pone.0107166.s001.pdf]

Supplementary Material: The effect of freeze-thaw cycles on gene expression levels in lymphoblastoid cell lines

Minal Çalışkan, Jonathan K. Pritchard, Carole Ober, Yoav Gilad

Table of Contents

Figure S1. Study Design 2

Figure S2. Pair-wise Euclidean distances 3

Figure S3. Correlation between gene expression levels and EBV copy numbers 4

Figure S4. Relative mtDNA copy numbers in LCLs 5

Figure S5. Box plots of within-individual pair-wise correlation coefficients 6

Figure S6. PCA plots after regressing out the effects of EBV and mtDNA 7

Figure S7. Box plots of between-individual pair-wise correlation coefficients 8

Figure S8. Bar plots showing the percent genes with eQTLs 9

Figure S9. Mean coefficient of variation (CV) of gene expression 10

Figure S10. CV of gene expression in EBV and mtDNA regressed out data 11

Table S1. Properties of the cells used in the study 12

Table S2. *P* values derived from testing the hypothesis that the means of the EBV copy numbers between each freeze-thaw cycle are the same 13

Table S3. Pathway enrichment results of EBV correlated genes 14

Table S4. *P* values derived from testing the hypothesis that the means of the mtDNA copy numbers between each freeze-thaw cycle are the same 15

Table S5. *P* values derived from testing the hypothesis that the means of the inter-individual correlation between each freeze-thaw cycle are the same. 16

Table S6. Pathway enrichment results for the 'highly variable' genes across all cell types/freeze-thaw cycles 17

Table S7. Pathway enrichment results for the 'highly variable' genes only within a specific cell type/freeze-thaw cycle 18

Table S8. *P* values derived from testing the hypothesis that the proportion of genes with eQTLs among 'highly variable' genes is equal to that among all the genes in our data. 19

Table S9. *P* values derived from testing the hypothesis that the coefficient of variation of gene expression between genes with eQTLs is equal to that of all expressed genes 20

Table S10. Number of overlap between 503 genes that were 'highly variable' across each cell type/freeze-thaw cycle and genes with eQTLs. 21

Table S11. *P* values from the linear regressions testing the association between cell type, freeze-thaw cycle, individual and the first five principal components 22

Figure S1. A) CD20+ B cells and 6 independent LCL cultures were obtained from six unrelated individuals (3 males; M1, M2, M3, 3 females; F1, F2, F3) between October 2009 and January 2010. Each LCL culture was thawed at every 3 months (February 2011=cycle 1, June 2011=cycle 2, October 2011=cycle 3, February 2012=cycle 4, June 2012=cycle 5, and October 2012=cycle 6) and cultured until obtaining ~10 million cells. RNA and DNA were extracted at every other freeze-thaw cycle (June 2011=cycle 2, February 2012=cycle 4, October 2012=cycle 6). **B)** Genome wide gene expression data were obtained at 4 different time points. 47 technical hybridization replicates (rep) were included to be able to assess the reproducibility of the data.

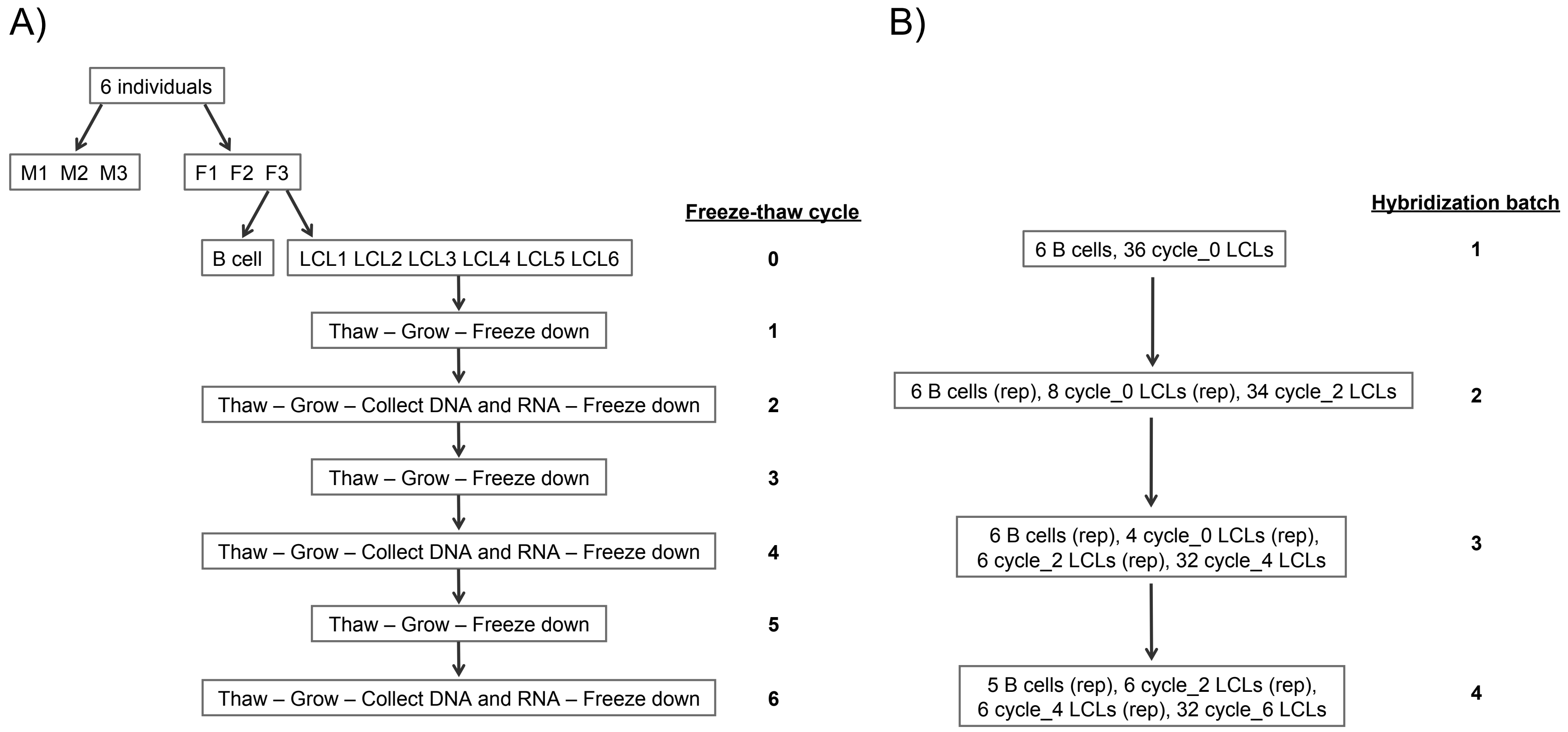

Figure S2. Pair-wise Euclidean distances along PC1/PC2 plane within and between classes of cells. T test on the pair wise Euclidean distance within and between classes of cells; $P < 2.2\times10^{-16}$.

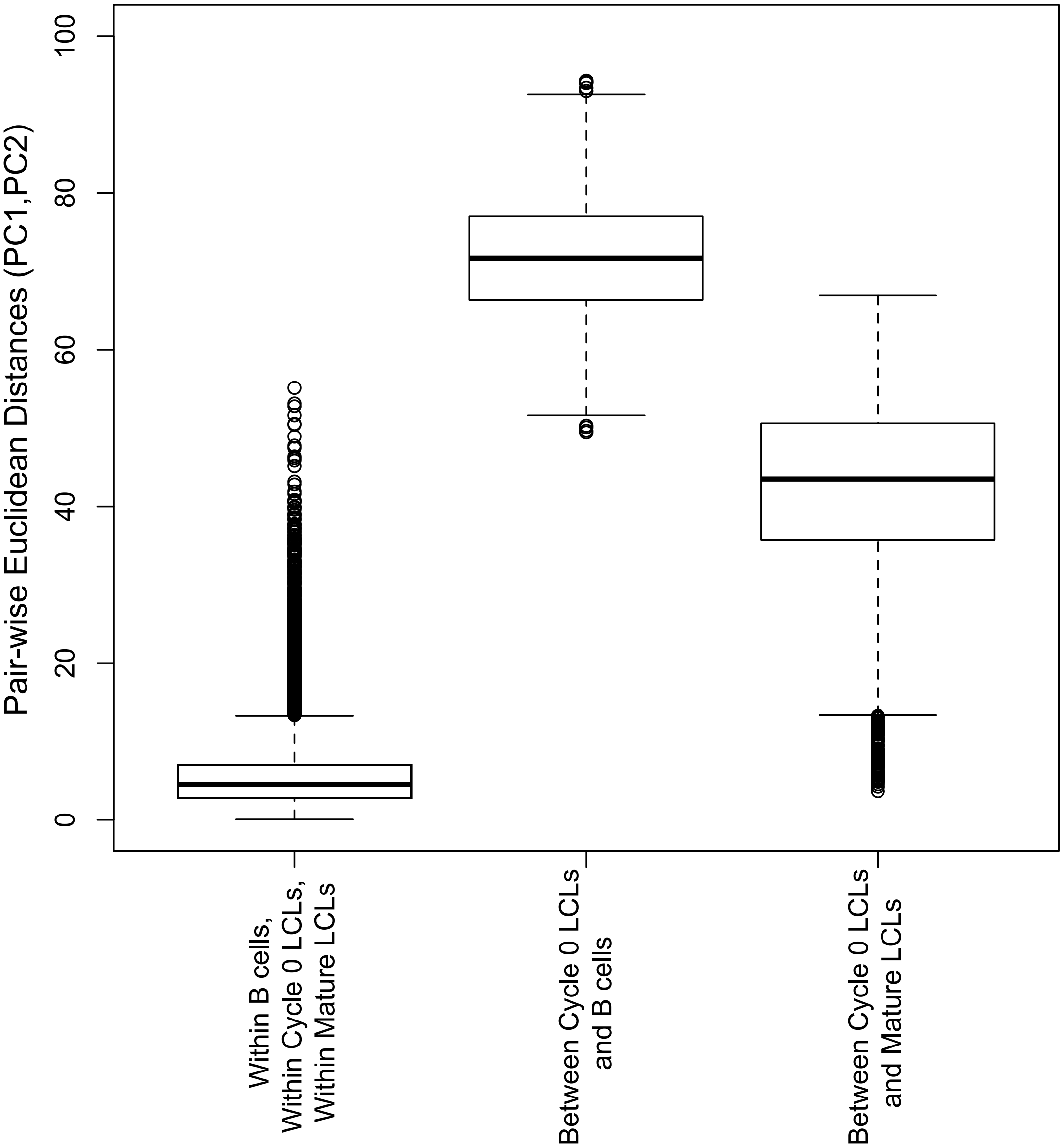

Figure S3. Distribution of the absolute value of Spearman rank correlation coefficient between gene expression levels and EBV copy numbers. Blue, green, purple, and orange curves correspond to correlation coefficients in cycle 0, cycle 2, cycle 4, and cycle 6 LCLs, respectively. Black, dark gray, and light gray curves correspond to each of the three null distributions within each cycle (expectation based on permutation).

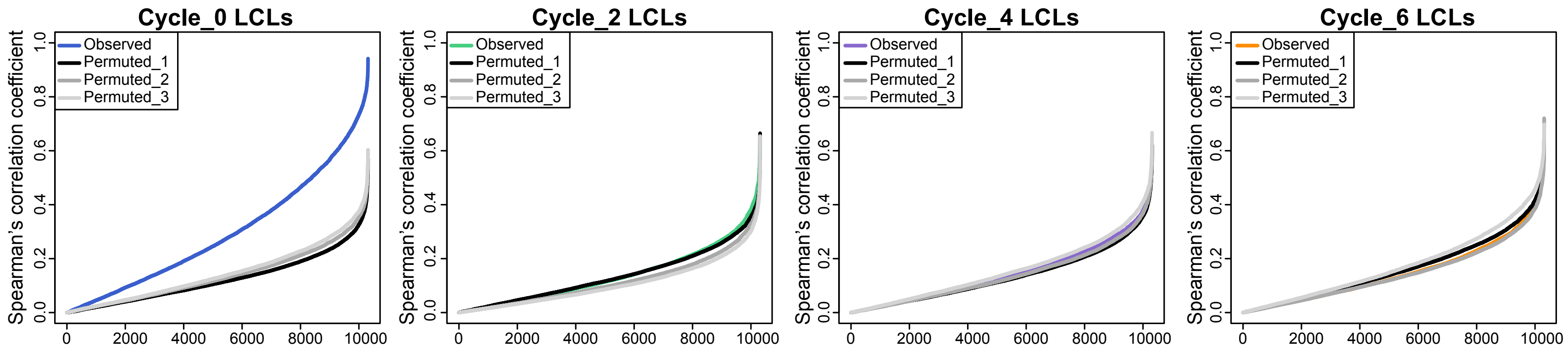

Figure S4. Relative mtDNA copy numbers in LCLs from six individuals. Error bars indicate the standard error of the mean.

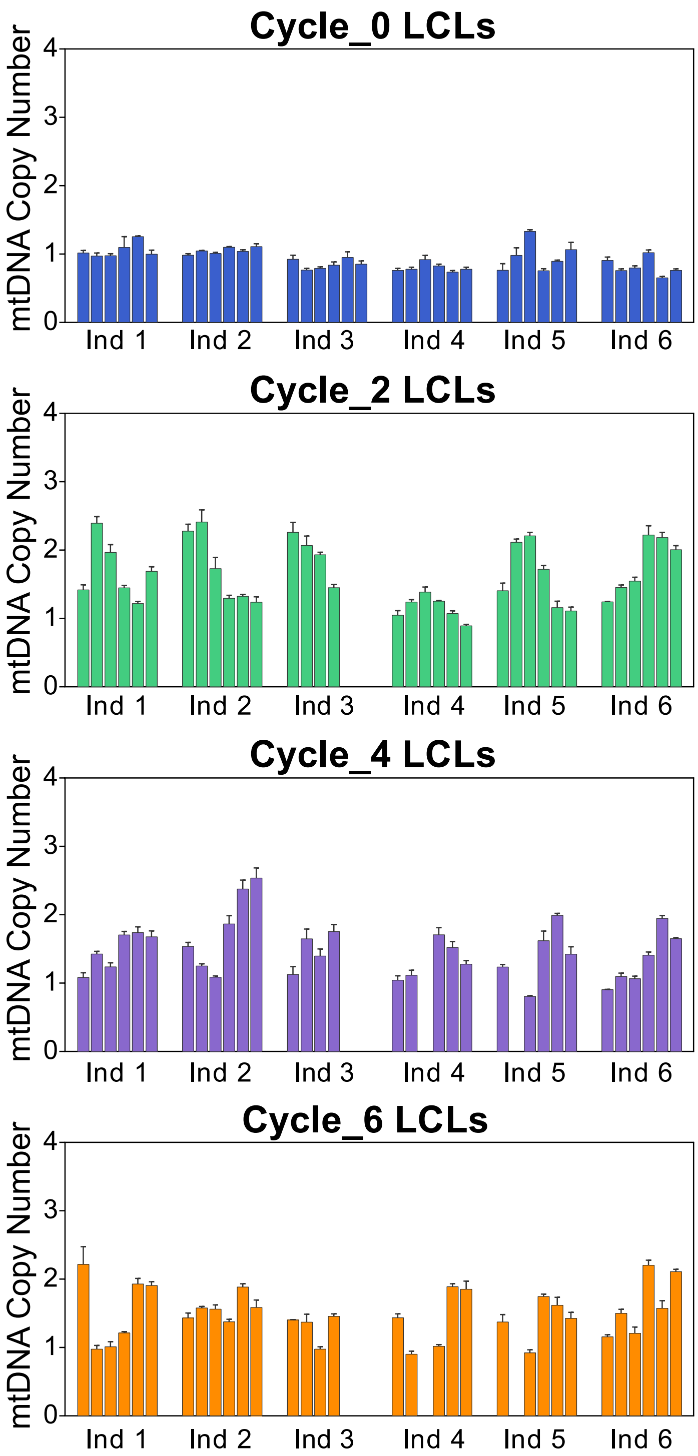

Figure S5. Box plots of within-individual pair-wise Pearson correlation coefficients of gene expression profiles in cycle 0 (c0), cycle 2 (c2), cycle 4 (c4), and cycle 6 (c6) LCLs.

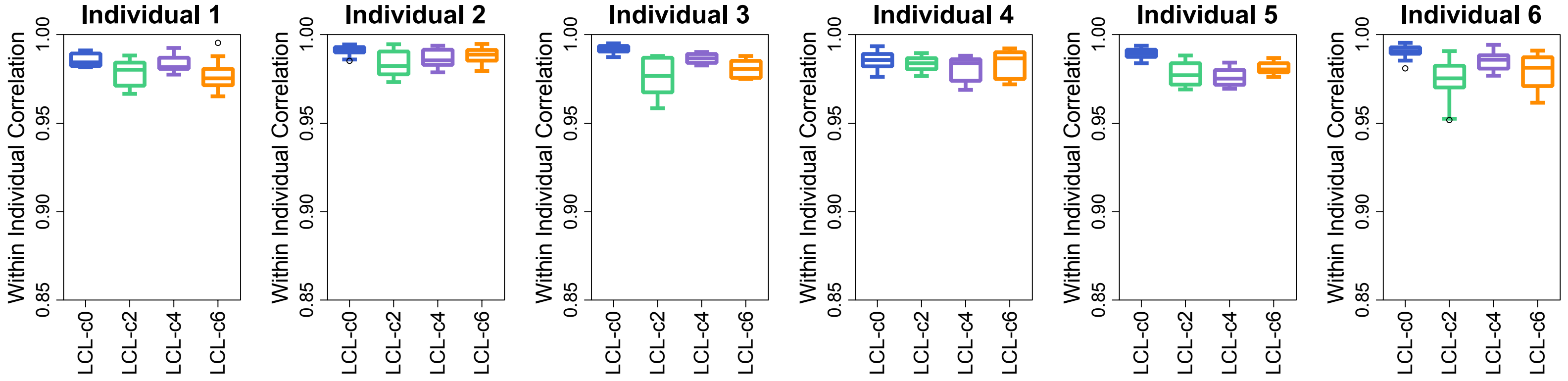

Figure S6. Plots of the first two principal components of the PCA after regressing out the effects of EBV and mtDNA copy numbers. PCA was applied to the genome-wide expression data of **A)** cycle 0 and cycle 2 **B)** cycle 0 and cycle 4 **C)** cycle 0 and cycle 6 LCLs. Numbers correspond to each of six individuals.

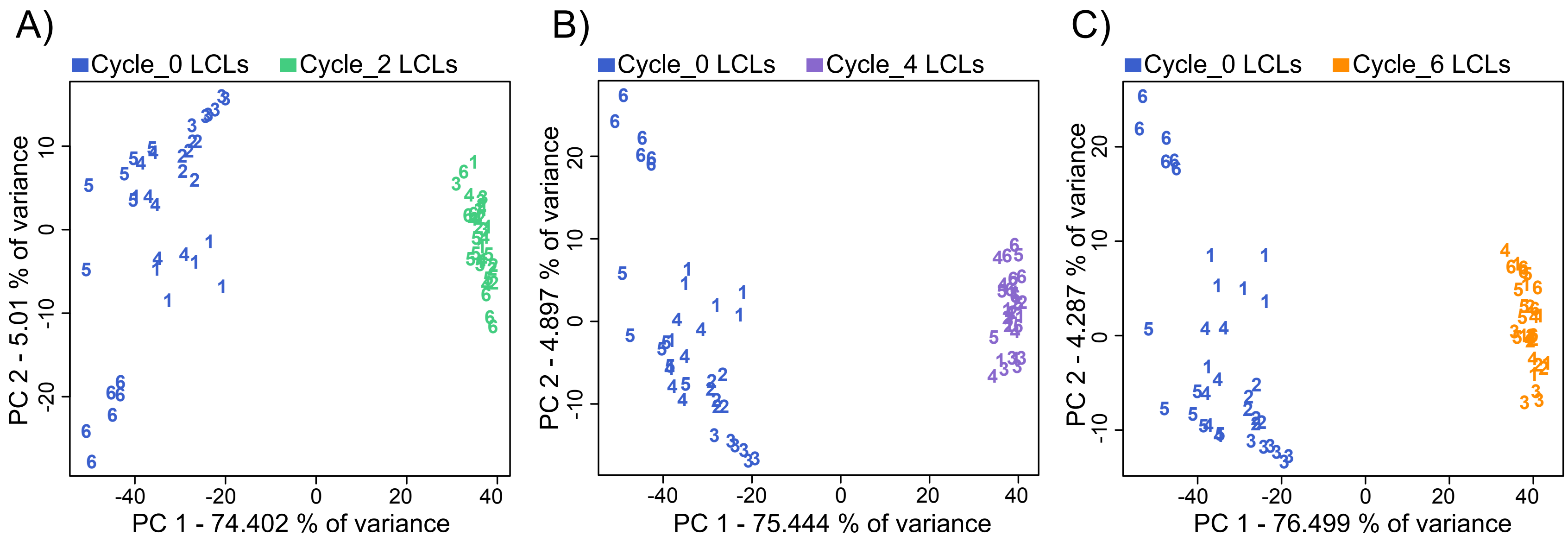

Figure S7. Box plots of between-individual pair-wise Pearson correlation coefficients of gene expression profiles in B cells, cycle 0 (c0), cycle 2 (c2), cycle 4 (c4), and cycle 6 (c6) LCLs**.**

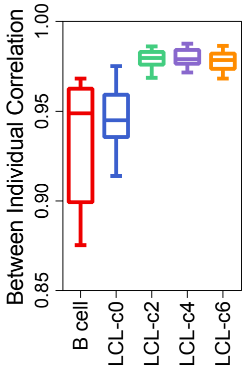

Figure S8. A) Bar plots showing the percent genes with eQTLs (as identified in seven eQTL studies performed in LCLs) among all the genes detected as expressed and among ‘highly variable’ genes in B cells, cycle 0, cycle 2, cycle 4, and cycle 6 LCLs. **B)** Bar plots showing the percent genes with eQTLs (as identified in eQTL studies in cortex, liver, fibroblast, T cell, and monocyte) among all the genes detected as expressed and among ‘highly variable’ genes in B cells, cycle 0, cycle 2, cycle 4, and cycle 6 LCLs. For each eQTL study, Fisher’s exact test (two-sided) was used to test the null hypothesis that the proportion of genes with eQTLs among ‘highly variable’ genes is equal to that among all the genes in our data. Asterisks indicate *P* values < 8.3×10^{-4} (Bonferroni corrected significance threshold). P values of each comparison are included in Table S8.

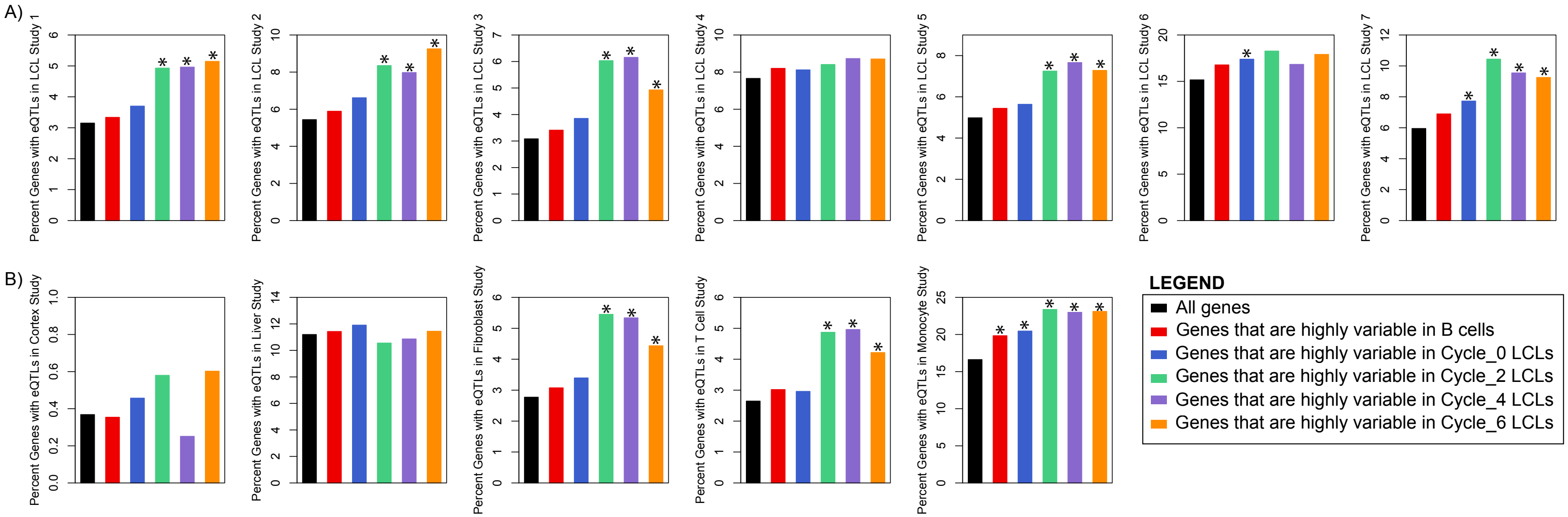

Figure S9. A) Mean coefficient of variation of gene expression within each cell type/freeze-thaw cycle for all the genes detected as expressed and for genes with eQTLs in seven different eQTL studies performed in LCLs. **B)** Mean coefficient of variation of gene expression within each cell type/freeze-thaw cycle for all the genes detected as expressed and for genes with eQTLs in eQTL studies performed in cortex, liver, fibroblast, T cell, and monocyte.

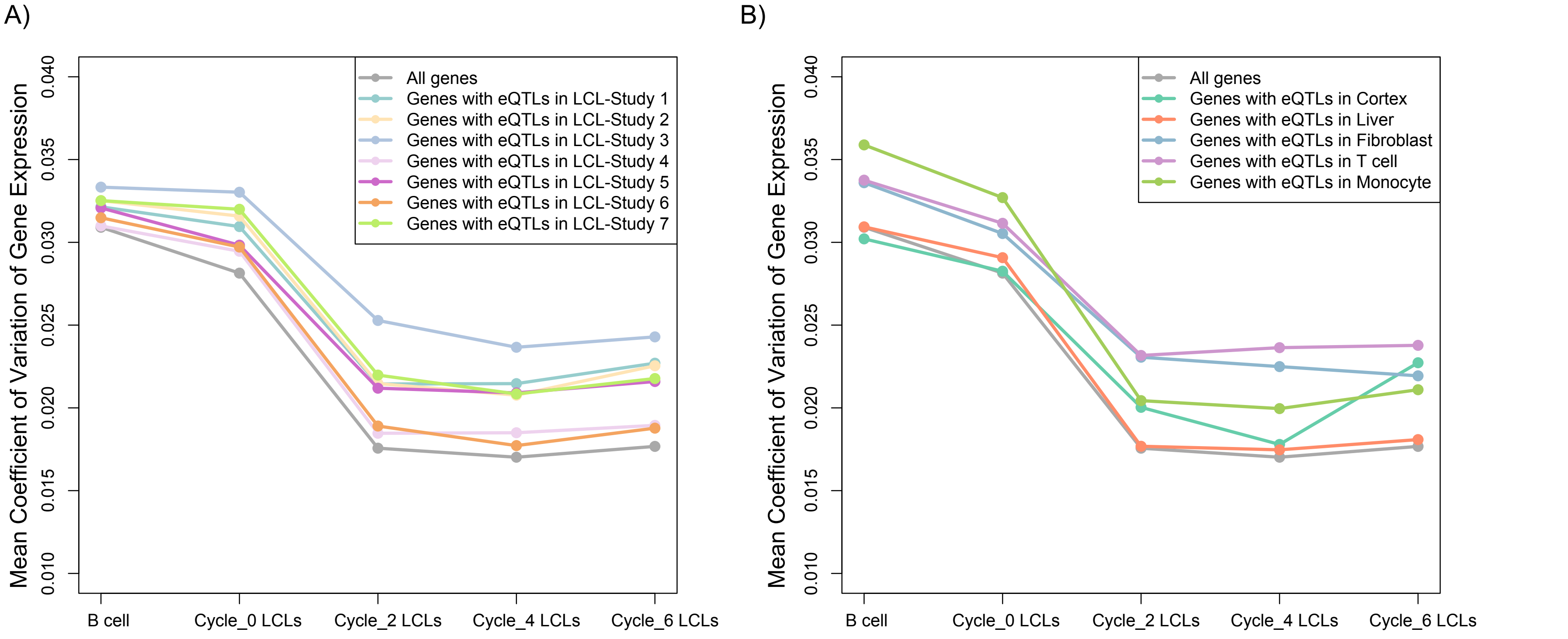

Figure S10. All results represented in Figure S10 are obtained after regressing out the effects of EBV and mtDNA copy numbers from gene expression data of each freeze-thaw cycle. **A)** Density distributions of the coefficient of variation (CV) of gene expression between-individuals within primary B cells and within LCLs of each freeze-thaw cycle. Black vertical line designates the arbitrarily chosen threshold of CV of 0.025. **B)** Bar plots showing the numbers of genes classified as having 'highly variable' expression patterns in the primary B cells, cycle 0, cycle 2, cycle 4, and cycle 6 LCLs. **C)** Venn diagram showing the overlaps in genes with 'highly variable' expression patterns across primary B cells, cycle 0, cycle 2, cycle 4, and cycle 6 LCLs.

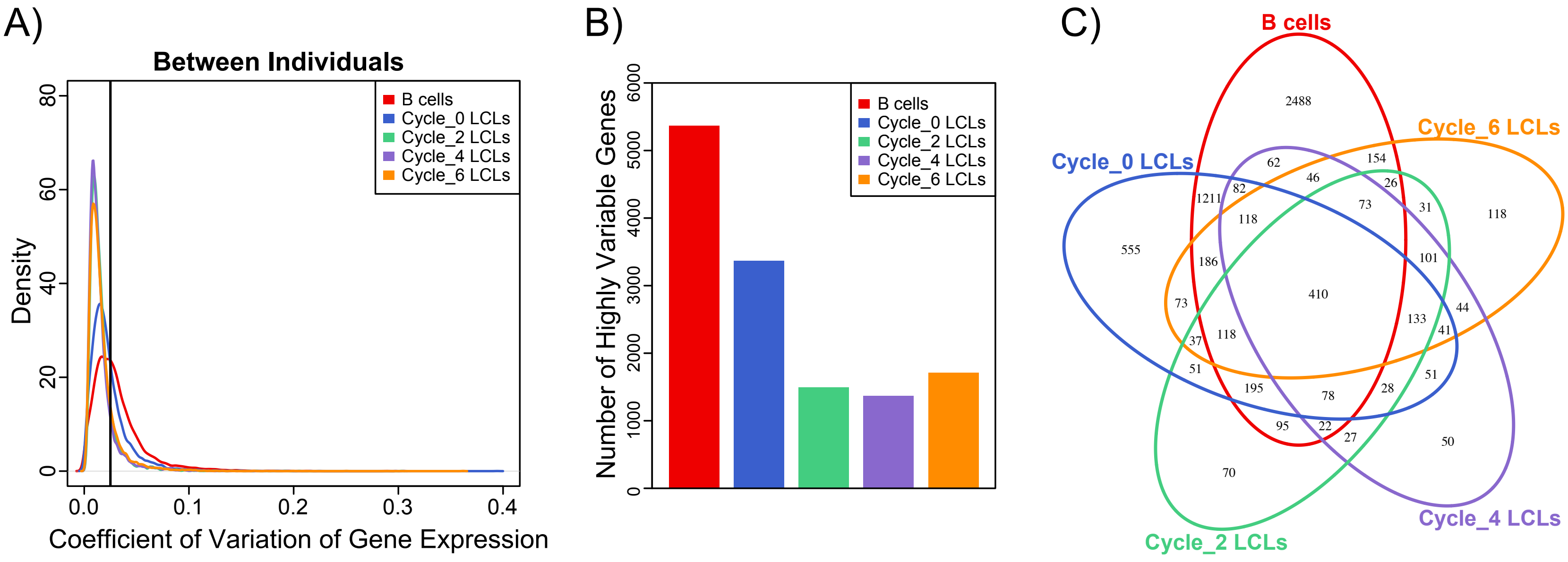

Table S1. Properties of the cells used in the study. For CD20+ B cells, time period of cell isolation/culturing corresponds to the dates when the cells were isolated. For Cycle 0 LCLs, time period of cell isolation/culturing corresponds to the dates from LCL establishment to freezing-down. For the rest of the LCLs, time period of cell isolation/culturing corresponds to the dates from thawing the LCLs to freezing-down. Number of freeze-thaw cycles is the number of times cells were frozen-thawed prior to cell culturing. Passage number refers to the number of times that a cell population has been transferred from one cell culture flask to another. RNA/DNA and gene expression data collection were performed at every other freeze-thaw cycle.

Cell Type	Time period of cell isolation/culturing	Number of freeze-thaw cycles	Passage number	RNA/DNA collection	Gene expression data collection
CD20+ B cells	Oct 09 - Jan 10	0	0	Yes	Yes
Cycle_0 LCLs	Oct 09 - Feb 10	0	1	Yes	Yes
Cycle_1 LCLs	Feb 11 - May 11	1	3	No	No
Cycle_2 LCLs	June 11 - Aug 11	2	5	Yes	Yes
Cycle_3 LCLs	Nov 11 - Dec 11	3	7	No	No
Cycle_4 LCLs	Feb 12 - Apr 12	4	9	Yes	Yes
Cycle_5 LCLs	June 12 - Aug 12	5	11	No	No
Cycle_6 LCLs	Oct 12 - Dec 12	6	13	Yes	Yes

Table S2. *P* values derived from testing (T-test; two-sided) the null hypothesis that the means of the EBV copy numbers between each freeze-thaw cycle comparison are the same.

**Comparison**	***P* value**
Cycle_0 vs. Cycle_2	4.94×10^{-05}
Cycle_0 vs. Cycle_4	2.23×10^{-06}
Cycle_0 vs. Cycle_6	2.65×10^{-05}
Cycle_2 vs. Cycle_4	0.7273
Cycle_2 vs. Cycle_6	0.1355
Cycle_4 vs. Cycle_6	0.04081

Table S3. Kyoto Encyclopedia of Genes and Genomes (KEGG) pathway enrichment results for the 1,719 genes whose expression was correlated with EBV copy number among cycle 0 LCLs. Enriched KEGG pathways at *P* value cut-off of 0.1 are shown.

**Enriched KEGG pathway**	**Fold enrichment**	**Enrichment *P* value**	**Enrichment *P* value (FDR-adjusted)**
DNA replication	3.4	1.8×10^{-6}	3.2×10^{-4}
Cell cycle	2.1	7.0×10^{-6}	6.3×10^{-4}
Parkinson's disease	2.1	1.2×10^{-4}	7.2×10^{-3}
Base excision repair	2.9	5.5×10^{-4}	2.5×10^{-2}
Oxidative phosphorylation	1.9	2.8×10^{-3}	9.8×10^{-2}
Huntington's disease	1.6	5.4×10^{-3}	1.5×10^{-1}
Homologous recombination	2.6	6.7×10^{-3}	1.6×10^{-1}
Alzheimer's disease	1.6	9.7×10^{-3}	2.0×10^{-1}
Pyrimidine metabolism	1.6	1.9×10^{-2}	3.2×10^{-1}
Oocyte meiosis	1.6	3.1×10^{-2}	4.4×10^{-1}
Mismatch repair	2.5	4.8×10^{-2}	5.5×10^{-1}
Valine, leucine and isoleucine degradation	1.9	5.4×10^{-2}	5.6×10^{-1}
RIG-I-like receptor signaling pathway	1.6	5.6×10^{-2}	5.5×10^{-1}
Fatty acid elongation in mitochondria	3.5	8.6×10^{-2}	6.9×10^{-1}

Table S4. *P* values derived from testing (T-test; two-sided) the null hypothesis that the means of the mtDNA copy numbers between each freeze-thaw cycle comparison are the same.

Comparison	*P* value
Cycle_0 vs. Cycle_2	$9.72x10^{-11}$
Cycle_0 vs. Cycle_4	$7.53x10^{-09}$
Cycle_0 vs. Cycle_6	$6.02x10^{-10}$
Cycle_2 vs. Cycle_4	0.153
Cycle_2 vs. Cycle_6	0.19
Cycle_4 vs. Cycle_6	0.8595

Table S5. *P* values derived from testing (T-test; two-sided) the null hypothesis that the means of the inter-individual correlation between each freeze-thaw cycle comparison are the same.

Comparison	P value
Cycle_0 vs. Cycle_2	$2.66x10^{-06}$
Cycle_0 vs. Cycle_4	$1.98x10^{-06}$
Cycle_0 vs. Cycle_6	$4.25x10^{-06}$
Cycle_2 vs. Cycle_4	0.6308
Cycle_2 vs. Cycle_6	0.5222
Cycle_4 vs. Cycle_6	0.2556

Table S6. KEGG pathway enrichment results for the 503 ‘highly variable’ genes across all cell types/freeze-thaw cycles. Enriched KEGG pathways at *P* value cut-off of 0.1 are shown.

**Enriched KEGG pathway**	**Fold enrichment**	**Enrichment *P* value**	**Enrichment *P* value (FDR-adjusted)**
Chemokine signaling pathway	2.1	6.0×10^{-3}	5.7×10^{-1}
Axon guidance	2.6	7.2×10^{-3}	4.0×10^{-1}
Jak-STAT signaling pathway	2.2	1.1×10^{-2}	4.0×10^{-1}
Cytokine-cytokine receptor interaction	2.0	1.5×10^{-2}	4.2×10^{-1}
Viral myocarditis	3.0	2.4×10^{-2}	5.0×10^{-1}
B cell receptor signaling pathway	2.4	3.2×10^{-2}	5.3×10^{-1}
ErbB signaling pathway	2.3	3.5×10^{-2}	5.1×10^{-1}
Thyroid cancer	3.5	4.8×10^{-2}	5.8×10^{-1}
Bladder cancer	2.9	5.0×10^{-2}	5.5×10^{-1}
Hematopoietic cell lineage	2.5	5.8×10^{-2}	5.7×10^{-1}
Natural killer cell mediated cytotoxicity	2.0	6.2×10^{-2}	5.6×10^{-1}
Leukocyte transendothelial migration	2.0	7.0×10^{-2}	5.7×10^{-1}
Acute myeloid leukemia	2.3	7.6×10^{-2}	5.7×10^{-1}
MAPK signaling pathway	1.6	8.2×10^{-2}	5.8×10^{-1}
Phosphatidylinositol signaling system	2.2	8.9×10^{-2}	5.8×10^{-1}
Glycine, serine and threonine metabolism	3.7	9.0×10^{-2}	5.6×10^{-1}
Intestinal immune network for IgA production	2.8	9.9×10^{-2}	5.8×10^{-1}

Table S7. KEGG pathway enrichment results for the 'highly variable' genes only within a specific cell type/freeze-thaw cycle. Enriched KEGG pathways at *P* value cut-off of 0.1 are shown. Results shown for **A)** 1,862 'highly variable' genes only in B cells **B)** 891 'highly variable' genes only in cycle 0 LCLs **C)** 101 'highly variable' genes only in cycle 6 LCLs. There were no enriched pathways among 74 'highly variable' genes only in cycle 2 LCLs and 49 'highly variable' genes only in cycle 4 LCLs at *P* value cut-off of 0.1

A) 1,862 'highly variable' genes only in B cells

Enriched KEGG pathway	Fold enrichment	Enrichment *P* value	Enrichment *P* value (FDR-adjusted)
Ubiquitin mediated proteolysis	1.6	$6.9x10^{-3}$	$6.9x10^{-1}$
Spliceosome	1.6	$1.9x10^{-2}$	$8.0x10^{-1}$
TGF-beta signaling pathway	1.8	$5.4x10^{-2}$	$9.6x10^{-1}$
mTOR signaling pathway	1.8	$5.4x10^{-2}$	$9.6x10^{-1}$
Apoptosis	1.5	$7.2x10^{-2}$	$9.6x10^{-1}$
Glycerophospholipid metabolism	1.8	$9.3x10^{-2}$	$9.6x10^{-1}$

B) 891 'highly variable' genes only in cycle 0 LCLs

Enriched KEGG pathway	Fold enrichment	Enrichment *P* value	Enrichment *P* value (FDR-adjusted)
Cell cycle	2.2	$3.1x10^{-3}$	$3.8x10^{-1}$
Aminoacyl-tRNA biosynthesis	2.9	$9.3x10^{-3}$	$5.2x10^{-1}$
RNA degradation	2.2	$7.1x10^{-2}$	$9.8x10^{-1}$
Terpenoid backbone biosynthesis	3.8	$8.2x10^{-2}$	$9.6x10^{-1}$
Spliceosome	1.6	$8.5x10^{-2}$	$9.4x10^{-1}$

C) 101 'highly variable' genes only in cycle 6 LCLs

Enriched KEGG pathway	Fold enrichment	Enrichment *P* value	Enrichment *P* value (FDR-adjusted)
Maturity onset diabetes of the young	34.8	$2.8x10^{-3}$	$1.8x10^{-1}$
Long-term potentiation	7.1	$6.1x10^{-2}$	$8.9x10^{-1}$
Gap junction	5.9	$8.5x10^{-2}$	$8.8x10^{-1}$
Melanogenesis	5.5	$9.7x10^{-2}$	$8.4x10^{-1}$

Table S8. *P* values derived from testing (Fisher's exact test; two-sided) the null hypothesis that the proportion of genes with eQTLs among 'highly variable' genes (HVG) is equal to that among all the genes in our data.

Comparison	LCL Study 1	LCL Study 2	LCL Study 3	LCL Study 4	LCL Study 5	LCL Study 6	LCL Study 7	Cortex Study	Liver Study	Fibroblast Study	T cell Study	Monocyte Study
HVG in B cells vs. All genes	0.5352	0.2415	0.2712	0.2343	0.2226	8.96E-03	2.20E-02	1	0.6703	0.2904	0.1828	6.96E-07
HVG in Cycle_0 LCLs vs. All genes	0.09222	4.98E-03	1.74E-02	0.3376	0.1009	6.16E-04	6.39E-05	0.4799	0.2095	0.04134	0.2779	1.97E-08
HVG in Cycle_2 LCLs vs. All genes	3.18E-04	5.11E-06	1.05E-08	0.2849	1.95E-04	0.001245	5.89E-11	0.2124	0.4561	5.89E-08	2.38E-06	3.75E-11
HVG in Cycle_4 LCLs vs. All genes	4.43E-04	1.06E-04	1.07E-08	0.1453	2.56E-05	0.0862	2.22E-07	0.649	0.7322	3.58E-07	2.22E-06	1.51E-09
HVG in Cycle_6 LCLs vs. All genes	4.96E-05	2.40E-09	1.31E-04	0.1301	1.15E-04	0.003269	4.74E-07	0.1587	0.7479	2.64E-04	4.52E-04	7.13E-11

Table S9. *P* values derived from testing (T-test; two-sided) the null hypothesis that the mean of the coefficient of variation of gene expression between genes with eQTLs is equal to that of all the genes detected as expressed.

Comparison	B cell	Cycle_0 LCLs	Cycle_2 LCLs	Cycle_4 LCLs	Cycle_6 LCLs
Genes with eQTLs in LCL-Study 1 vs. All genes	0.3912	0.05667	0.003533	0.001108	0.0002601
Genes with eQTLs in LCL-Study 2 vs. All genes	0.1232	0.001252	$1.45x10^{-05}$	$1.90x10^{-05}$	$3.09x10^{-07}$
Genes with eQTLs in LCL-Study 3 vs. All genes	0.112	0.001431	$6.95x10^{-07}$	$8.04x10^{-06}$	$9.57x10^{-06}$
Genes with eQTLs in LCL-Study 4 vs. All genes	0.9207	0.09385	0.1555	0.02411	0.05787
Genes with eQTLs in LCL-Study 5 vs. All genes	0.3	0.123	0.0001259	$6.62x10^{-05}$	$8.25x10^{-05}$
Genes with eQTLs in LCL-Study 6 vs. All genes	0.2948	0.003052	0.0002894	0.03685	0.003905
Genes with eQTLs in LCL-Study 7 vs. All genes	0.0555	$5.13x10^{-06}$	$9.43x10^{-12}$	$1.97x10^{-09}$	$2.68x10^{-09}$
Genes with eQTLs in Cortex vs. All genes	0.8558	0.9705	0.3255	0.7476	0.1098
Genes with eQTLs in Liver vs. All genes	0.9708	0.1654	0.8063	0.3142	0.4065
Genes with eQTLs in Fibroblast vs. All genes	0.06161	0.05469	$1.90x10^{-06}$	$3.05x10^{-06}$	$3.97x10^{-05}$
Genes with eQTLs in T cell vs. All genes	0.08497	0.0501	0.0002006	0.0001995	0.0001379
Genes with eQTLs in Monocyte vs. All genes	$7.32x10^{-14}$	$1.12x10^{-12}$	$1.02x10^{-09}$	$1.96x10^{-09}$	$2.84x10^{-12}$

Table S10. Number of overlap between 503 genes that were 'highly variable' across each cell type/freeze-thaw cycle and genes with eQTLs (as identified in each of the eQTL study). Hypergeometric test is used to calculate the significance of overlap between 503 'highly variable' genes across each cell type/freeze-thaw cycle and genes with cis eQTLs that were detected as expressed in our study.

eQTL study name	Number of Ensembl genes with cis eQTLs	Number of Ensembl genes with cis eQTLs that were detected as expressed in our study	Number of overlap between 503 genes that were 'highly variable' across each cell type/freeze-thaw cycle and genes with cis eQTLs	*P* value (Significance of overlap)
LCL-Study 1	438	325	26	0.0090
LCL-Study 2	794	561	49	4.63×10^{-05}
LCL-Study 3	442	318	42	3.00×10^{-09}
LCL-Study 4	1084	790	47	0.088
LCL-Study 5	830	514	53	1.50×10^{-07}
LCL-Study 6	1894	1564	93	0.021
LCL-Study 7	778	614	57	1.63×10^{-06}
Cortex-Study	68	38	2	0.56
Liver-Study	2057	1155	56	0.54
Fibroblast-Study	427	286	32	9.31×10^{-06}
T cell-Study	430	273	30	2.51×10^{-05}
Monocyte-Study	2510	1714	138	2.64×10^{-10}

Table S11. *P* values from the linear regressions testing the association between three variables (cell type, freeze-thaw cycle, individual) and the axis-scores of the first five principal components **A)** PCA was applied to 10,313 genes that were detected as expressed. **B)** PCA was applied to 26, 49, 42, 47, 53, 93, and 57 genes that were both among the 'highly variable' genes across all cell types/freeze-thaw cycles in our study and that had eQTLs in the corresponding eQTL studies (in LCLs). **C)** PCA was applied to 2, 56, 32, 30, and 138 genes that were both among the 'highly variable' genes across all cell types/freeze-thaw cycles in our study and that had eQTLs in the corresponding eQTL studies (in cortex, liver, fibroblast, T cell, and monocyte).

A)

Principal Components	10,313 Genes		
	**Cell type**	**Freeze-thaw cycle**	**Individual**
PC1	9.192E-14	0.000000719	0.9999
PC2	0.05953	0.03997	0.9986
PC3	0.7296	0.6477	0.4433
PC4	0.8516	0.002356	0.4261
PC5	0.8134	0.03624	0.03388

B)

Principal Components	26 Genes - LCL-Study 1		
	**Cell type**	**Freeze-thaw cycle**	**Individual**
PC1	0.01926	0.6678	0.000001762
PC2	1.279E-09	0.003237	0.5663
PC3	0.8518	0.7195	0.000001175
PC4	0.9605	0.04381	0.3094
PC5	0.6298	0.09555	0.4442
**Principal Components**	**49 Genes - LCL-Study 2**		
	**Cell type**	**Freeze-thaw cycle**	**Individual**
PC1	0.0005666	0.2653	0.0005349
PC2	0.000001117	0.001056	0.1112
PC3	0.3536	0.004043	0.9575
PC4	0.7508	0.655	0.01657
PC5	0.7039	0.5626	0.0003505
**Principal Components**	**42 Genes - LCL-Study 3**		
	**Cell type**	**Freeze-thaw cycle**	**Individual**
PC1	0.006844	0.4172	0.00002487
PC2	0.000002158	0.001768	0.1231
PC3	0.2309	0.0445	0.5274
PC4	0.1325	0.6045	0.00000527
PC5	0.6214	0.4274	0.000001291
**Principal Components**	**47 Genes - LCL-Study 4**		
	**Cell type**	**Freeze-thaw cycle**	**Individual**
PC1	0.001035	0.2641	0.0002442
PC2	0.000003325	0.001011	0.1346
PC3	0.1547	0.03196	0.6482
PC4	0.7822	0.03547	0.02793
PC5	0.9387	0.8243	0.03638
**Principal Components**	**53 Genes - LCL-Study 5**		
	**Cell type**	**Freeze-thaw cycle**	**Individual**
PC1	3.122E-08	0.0001649	0.2953
PC2	0.08875	0.1081	0.00007801

PC3	0.02251	0.2141	0.7265
PC4	0.8947	0.5244	0.001113
PC5	0.6212	0.5445	0.02624

Principal Components	93 Genes - LCL-Study 6		
	Cell type	Freeze-thaw cycle	Individual
PC1	6.313E-14	0.000001159	0.9965
PC2	0.09683	0.03447	0.9871
PC3	0.9226	0.02706	0.05109
PC4	0.6905	0.9664	0.3217
PC5	0.8692	0.7758	0.000003368

Principal Components	57 Genes - LCL-Study 7		
	Cell type	Freeze-thaw cycle	Individual
PC1	5.327E-14	0.0000326	0.9569
PC2	0.4234	0.5526	1.715E-07
PC3	0.3991	0.02191	0.4518
PC4	0.5794	0.7388	0.005308
PC5	0.3361	0.7571	0.002215

C)

Principal Components	2 Genes - Cortex Study		
	Cell type	Freeze-thaw cycle	Individual
PC1	0.08494	0.3628	3.539E-07
PC2	0.9996	0.2712	0.01317
PC3	NA	NA	NA
PC4	NA	NA	NA
PC5	NA	NA	NA

Principal Components	56 Genes - Liver Study		
	Cell type	Freeze-thaw cycle	Individual
PC1	2.901E-16	0.00003945	0.9985
PC2	0.1722	0.05871	0.8866
PC3	0.7448	0.02392	0.06088
PC4	0.8004	0.611	0.000009785
PC5	0.9919	0.6978	0.04852

Principal Components	32 Genes - Fibroblast Study		
	Cell type	Freeze-thaw cycle	Individual
PC1	< 2.2e-16	0.000167	0.9959
PC2	0.9893	0.2143	0.00009866
PC3	0.51	0.05103	0.06856
PC4	0.8559	0.7027	0.001544
PC5	0.7647	0.2029	0.01543

Principal Components	30 Genes - T cell Study		
	Cell type	Freeze-thaw cycle	Individual
PC1	0.07886	0.9375	0.000000496
PC2	1.043E-11	0.00421	0.5823
PC3	0.4782	0.7275	0.000002201
PC4	0.9457	0.01068	0.1195
PC5	0.7094	0.6706	4.185E-09

Principal Components	138 Genes - Monocyte Study		
	Cell type	Freeze-thaw cycle	Individual
PC1	1.88E-13	9.906E-07	0.9969
PC2	0.7359	0.9613	1.855E-08
PC3	0.08609	0.02456	0.9387
PC4	0.4502	0.05058	0.2185
PC5	0.7987	0.5179	0.04216
